# The Röntgen Ray

**Published:** 1896-12

**Authors:** William Lincoln Smith


### International Dental Journal.

Vol. XVII. December, 1896. No. 12.

### Original Communications.[1]

### THE RÖNTGEN RAY.[2]

BY WILLIAM LINCOLN SMITH.[3]

I shall probably have comparatively little to say in the course of my talk which has not appeared in one form or another in some of the technical journals or some portions of the daily press. Nevertheless, there has been a great deal of misunderstanding on the subject, and conflicting and contradictory reports have arisen, simply on account of the enthusiasm of the reporters, especially of that portion of the press which reaches that class of people who at present take no particular interest in this matter. In order that the subject may be clear, it seems well to go back a little that we may see how the Röntgen rays are related to some of the phenomena of electrical science.

Of course, the easiest form in which to illustrate the character of the electric spark is the common discharge between the poles of a static or influence machine, the apparatus which we use to explain the phenomenon of the lightning flash. Now, when that discharge

[1] The editor and publishers are not responsible for the views of authors of papers published in this department, nor for any claim to novelty, or otherwise, that may be made by them. No papers will be received for this department that have appeared in any other journal published in the country.

[2] A talk before the Harvard Odontological Society, May 28, 1896.

[3] Instructor in Electrical Engineering, Massachusetts Institute of Technology.

is allowed to pass from one pole to the other, the discharge is apparently the same in all its length; and as carefully as you may examine the terminals from which the discharge is passed, you will see no difference whatever between them, but if we make the experiment in perfect darkness and take a series of such photographs as those by Trouvellot, we shall find that there is a very distinct and characteristic difference between the two, according to which pole the discharge was derived from. This difference, of course, is very hard to define, and it is only by the aid of photographs that we are enabled to see that there is some difference there. Now, this discharge can be deflected, as you might expect, by passing the poles of a magnet near to it,—that is, the discharge sags under the pole of the magnet; but after it has dropped it returns to its original path, —it is only a momentary sag; there is no permanent deflection of the discharge by the action of the magnet.

Now, suppose we begin to send this discharge through atmosphere at a diminished pressure, in other words, through a greater or less vacuum. We shall find that the discharge has at first its ordinary thread-like form. As we increase the vacuum, we notice a difference in the appearance of the spark,—it begins to broaden; it will get "fatter," you might say, but at the same time that it increases in its diameter it will become fainter,—that is to say, it begins to approach more nearly to the form of a glow extending through the tube, but the light becomes paler, and finally we shall reach a point where we get what we might call billows of light, which run along from one terminal to the other. This is very characteristic of the electrical apparatus known as the Geissler tubes. The bands of light begin quite narrow and gradually change, and they become broader or narrower as you increase or diminish the vacuum. The point of interest does not lie in that breaking up of the discharge, but in the difference which begins to appear between the two poles. We will suppose that the discharge is made from the anode (which is the name given to the positive pole of an electrical battery or apparatus), and passes across the tube to the cathode (or the negative pole). We shall notice that one pole has a very brilliant, star-like effect at the very tip of the pole, which is usually made of some material suitable to the passage of electricity, we will say of aluminum wire, which is quite commonly used, and the size of it is, well, one-thirty-second of an inch in diameter; then at the very tip of that wire there would be a very brilliant, star-like point, from which a beautiful effect is derived. At the other pole we find the discharge is distributed more evenly over the whole surface of the wire; moreover, it does not appear to touch the wire. On close examination we shall find that there will be a very small space which is absolutely dark. It shows in the midst of the glow around the pole almost an inky blackness; there is absolutely nothing there that we can discover by the use of photography or any known method. It is right here that the subject begins to get interesting, for we find that the higher we increase the vacuum, the larger does that dark space become, and it is possible to obtain that dark space large enough to fill the whole tube in some cases. If you could obtain a complete vacuum, it would do so, but the tubes most commonly in use have about one-millionth of an atmosphere remaining (an atmosphere meaning fifteen pounds pressure), which is almost as low as it is possible to force the vacuum at present. Now, when we get that dark space expanded enough so as to include both the terminals, the tube becomes apparently impervious to the electric spark. It takes more power than we can derive from experimental machines to produce the results we desire, and we therefore have to use some more extensive apparatus for the generation of the necessary current, and to illustrate the effect of the vacuum on the electric current an apparatus has been devised which is so arranged that both poles are situated in the vacuum-tube within half an inch from each other; then the two electrodes are connected with a spiral tubing at points just before they enter the vacuum-tube, and the path around through that spiral tubing from one electrode to the other is something like two and one-half yards, while the distance across is only about half an inch. Now, it is well known that electricity in its passage from one point to another will take the path of least resistance; nevertheless, the discharge will pass every time through that tubing rather than pass from pole to pole in the vacuum-tube, simply because both terminals have their tips in the dark space.

When we have a vacuum-tube which shows quite a large dark space,—has a vacuum somewhere within one to two-millionths of an atmosphere pressure,—we have what is called a "Crookes tube," simply because Professor Crookes, of England, was prominently connected with the investigation of the high vacua phenommena; nevertheless, Hittorf, in Germany, was the first to study the peculiarities of the electric current in high vacua. Now, Professor Crookes found that there were many phenomena exhibited by the electric current in vacua that were not shown when the passage took place in the open air. First, he found that he had got rid entirely of all the effects that proceeded from the anode; secondly, the dark space around the cathode was enormously extended; and, thirdly, what we call the cathode rays were produced,—that is, when the discharge was allowed to pass from anode to cathode, apparently there were produced at the cathode another set of rays which travelled outward and passed in straight lines, and once deflected by the magnet did not return to their original path.

We shall see that there are a number of interesting phenomena produced by these cathode rays. For instance, if you interpose in the path of the rays a screen made of aluminum or platinum film, that film promptly casts its shadows on the wall of the tube; the cathode rays cause the interposed metal to glow with a brilliant light, which is called "luminescence;" it used to be called "fluorescence," but "luminescence" seems now to be the more scientific term for it. A great many substances are found to be luminescent to the cathode rays: most of the metals, aluminum, platinum, gold, silver, platino-barium cyanide; tungstate of calcium; a great many of the precious stones, diamonds, rubies, and emeralds, and these precious stones generally glow with most beautiful color; German glass glows with a bright apple-green. If, then, you place a film of any of the substances I have mentioned in between the source of the cathode rays and the wall of the tube, it will cause the substance to glow, and it will cast but little shadow on the wall of the tube; but, if you place a piece of mica or quartz in the same position, you will find that it will not light up so brilliantly, and that it will cast quite a dense shadow on the wall of the tube, showing that substances which are optically transparent are not necessarily so to the cathode rays. The explanation offered for this anomaly is that the cathode rays are not rays, as we understand the term when applied to rays of light, but a steady stream of molecules being carried along by the electric flow, since the vacuum is so high that what is called the "mean free path" for the molecules is sufficiently great to allow them to move clear across and strike against the wall of the tube, this wall thus being made luminescent by the steady molecular bombardment. That would seem to be rather a good explanation, because it is possible to make a toy wheel go round rapidly by the passage of the electric current through a vacuum tube, and it would seem as though the successive paddles were struck by this stream of molecules, causing it to revolve. Nevertheless, this theory was not received with great interest in Germany; it was the more common belief there that the cathode rays were some disturbance in the luminiferous ether,—they were not a bombardment of the molecules against the wall of the tube,—so that physicists there made many investigations and experiments in the endeavor to prove whether they were a molecular bombardment or not. Professor Hertz took up the subject (the same who enabled us finally, a few years ago, to measure the length of an electrical wave in space) and studied it quite carefully. He found that some substances put inside the Crookes tube were transparent to these rays and some were not, and, among other things, he found that certain metal films were transparent, provided you got them thin enough; that these rays would pass through certain thicknesses of gold, silver, copper, or platinum, yet they would not go through the same thickness of mica. Just when he reached this point he was led off from this line of investigation, and before he could take it up again he died. His assistant, Dr. Lenard, who has now taken his place at the Bonn University, took up the subject with the great desire of obtaining these cathode rays in the open air, where one could get at them and study them with less hinderance. To accomplish this he put a little window in his tube. As I have told you, Hertz had found that some substances were easily transparent to the cathode rays, and that of the metals aluminum was the most transparent,—that is to say, it was the metal which would allow the cathode rays to pass through in greatest volume; so Lenard made a small window in his Crookes tube of aluminum about three thirty-seconds of an inch or less wide. This was a very delicate piece of work, because if he had the aluminum too thick the cathode rays would not go through it as satisfactorily as he wanted, and if he had it too thin the atmospheric pressure would break the tube. Nevertheless, he succeeded in getting the cathode rays to come out into the open air. Immediately on putting in his window and turning on the electric current he received a surprise which delighted him, for the whole air around the window for the distance of eight centimetres glowed with quite a bright purple glow, and he found that substances would luminesce outside of the tube in the air as well as inside, but the effect was not produced to any great extent and was absorbed by any great thickness of air. The rays outside of the tube could not traverse any great distance; nevertheless, they were deflected by the magnet and showed every characteristic of the cathode rays in the tube. He then attached to the end of his Crookes tube a long tube with a number of luminescent screens in it of varying density and arranged it so that he could vary the vacuum. He found that the higher he carried the vacuum the farther the cathode rays would pass, and that they would affect a photographic plate in the air a distance of about eight centimetres. There he stopped. He used his photographic plate, but he exposed the plate directly to the cathode rays. That was the only point that he differed from Professor Röntgen.

That was the point at which this matter stood last December when Professor Röntgen, who was also experimenting on these lines, made his great discovery by purely a matter of chance. He happened to be working with a tube that was covered with black paper. He had found that light had a bad effect on the tubes, and that they would deteriorate unless kept in darkness. He had just covered this tube with this black paper and turned on the current, when he noticed that a piece of platino-barium-cyanide paper which was near the tube glowed. Also, afterwards, in developing a photographic plate which had been lying near the tube on the table, he found not only the picture that he was working for on there, but also the image of a little brass spring on the plate-holder. How it came to get into the picture he could not understand, except that the plate in its holder was standing up near the tube at the time when he noticed the paper glowing, and it was in such a position that the spring was between the plate and the tube. Now, there was no window in this tube, so he knew that the cathode rays had not come out in their usual manner and affected the plate, but that it had been affected by some invisible agent. Of course that put him on the right track immediately, and it was not a very great while before he announced to the world that substances which had hitherto been considered opaque were transparent to the effects of an unknown agent which he called the "X-rays."

Just a word as to the apparatus with which Professor Röntgen conducted his experiments. He did not use any elaborate or specially designed apparatus, but simply a vacuum tube and an ordinary Rhumkorff induction coil. A current was set up in a small battery, causing a secondary spark to pass through the Crookes tube which was connected to the poles of the induction coil; the tube was illuminated, and of course produced its effects. He studied the source of the rays very thoroughly,—in fact, he made a very beautiful investigation before he mentioned anything about it at all, and there is hardly a thing been since noted with regard to their effects which was not at least suggested if not tried by him. It was a most thorough investigation. Among other things, he examined the source of the rays, and while he could make no positive statement as to the exact manner in which they were produced, he declared that they could not by any possibility be the cathode rays: first, because the Röntgen rays can never be deflected, the most powerful magnetic fields that we can produce have apparently no effect in turning them from straight lines; secondly, the Röntgen rays travel enormous distances, whereas the cathode rays do not reach more than eight centimetres. The effect of the Röntgen rays has been traced through six men,—that is to say, the testing apparatus would be affected beyond and through that thickness of flesh.

As soon as Röntgen published his paper, the investigation was taken up by physicists all over the world with a great deal of energy. One of the first questions which they sought to find some answer for was, What was the source of the rays? This question has not yet been satisfactorily answered, as their origin cannot be traced beyond the luminescent spot on the tube. The point on the tube where the cathode rays struck and produced their brilliant green luminescence was apparently the source of the rays, but, as I have said before, they were not the cathode rays. He called them the "X-rays." Some of the investigators thought that these rays were not related to the cathode rays; Professor Rowland, for instance, claiming that they proceeded from the anode. Professor Elihu Thomson also thought so, but later recanted, and said he did not think that was the fact. Rowland afterwards said that he would prefer to investigate further before making any statements as to what he thought was the source of the rays. Experiments were continued, and it has finally been settled upon that the Röntgen rays are produced at the first point that intercepts the cathode rays.

Now, the Röntgen rays had been excited by means of the ordinary Rhumkorff coil, and it was not long before we found that that had an effect of heating the tube, which apparently had a deleterious effect as regards the production of these rays, and it was only a brief time before the tube would be destroyed. Some years ago Professor Thomson and Tesla were both experimenting in the same line, with what is called the high frequency alternating current, which oscillates something like a million times a second, and that discharge, at high frequency, is perfectly innocuous to the human body. You could receive a spark on your hand and you would never know it touched you, so that we thought possibly that with that very high oscillation we could get a current which would excite the tube and not produce these unfortunate heating effects. That proved to be so, and, moreover, as we could get a great deal of electrical energy into the tube without damaging it, we had more powerful effects from the rays and more distinct photographs.

Coming down, then, to the static influence-machine, which is something like the common frictional machine, except that the current is not produced by friction but by induction, we secured even better results with that than with the high-frequency current. A photograph which we could take in perhaps ten minutes with the friction-machine, we could take in a few seconds with the influence-machine. This influence-machine is driven, generally, by a small motor, though the power may be obtained in various ways, and we allow its spark to pass through the tube from anode to cathode, and under certain conditions (which are not ready for publication until the system has been thoroughly tried), when it is wired in a certain way, we seem to get very much more powerful effects than we do in any other way.

We come now to the examining or testing apparatus by which the effects of these invisible rays are made visible to us. One of the simplest tests is the photograph. If, for instance, you wish to take a photograph of the hand, you put a sufficiently sensitive plate in a plate-holder and place your hand on the cover, turn on the rays, and you will get a photograph of the bones and denser tissues of the hand, simply because the ability of the rays to penetrate substances varies almost directly as the density of the substances through which the rays are passing. The photograph, however, is open to certain objections. In the first place, it requires a certain amount of time to take and develop the picture; and, secondly, it furnishes a permanent record, which in some cases you do not want, as your picture may not prove to contain what you are looking for, and this method is not quite so satisfactory as if you could examine the object itself instead of the photograph of it. Mr. Edison has made this possible by the invention of the fluoroscope, which is a large pyramidal box built after the plan of those stereoscopes that you view by reflected light, only in place of the ground glass screen at the larger end of it, there is a paper which is coated with a surface of tungstate of calcium. Now, tungstate of calcium can be prepared either by crystallization or fusion. When prepared chemically, by crystallization, it does not luminesce at all, and is perfectly worthless for the purpose for which we need it; when it is prepared by fusion in a furnace, it will luminesce with the most astounding brilliancy. That piece of paper is put in the box, and then if we wish to make an examination of the hand, we should put the Crookes tube here and hold the hand up and look through the fluoroscope, and we see on the illuminated screen beyond a shadow picture, portions of the shadow appearing darker or lighter according to the density of the tissue through which the rays have to pass. The photographs are not to be compared with the picture as seen in this way; you will see not only every bone and its joints, but when properly adjusted you can see the cartilage, the folds in the skin, the lines of the cords, and even the nerves sometimes, and all with the most wonderful clearness. We have succeeded in forcing the rays through the body sufficiently to enable us to determine the exact location and size of the various organs, and the subject has been of the deepest interest to those who, at the Institute, have been working with Mr. Norton, one of the instructors in physics there. I, myself, have not been working very much with the rays, being engaged in another line of study at present; but I have endeavored to keep posted on the experiments and improvements that have been made by others. They have been able to see, with the aid of the rays, the condition of the lungs of a person in advanced consumption; have traced out with perfect clearness on that screen the diseased portion,—it can be distinguished by its absolute opacity compared with the healthy tissue, which is quite transparent. A case of enlarged spleen has shown up quite satisfactorily; you can see the liver rise and fall, and determine the exact position of the organs within one-sixteenth of an inch; you can just see the tip of the kidney, which is apparently more opaque and denser; the liver rises and falls in front of it as the patient breathes; you can see the ribs, which look like the boards of a gate; you can see the spine with its many joints; you can see the sharp definition of the teeth as they run down into the jaws, and the limits of a filling that is put into a tooth. We are still trying to get more sensitiveness and power in the apparatus in order to make visible a great many things which do not show up now.

I have brought down a great number of photographs which will serve to illustrate the various steps which we have gone through from our first efforts, requiring several minutes, to the present system by which a photograph may be taken in a few seconds. This first one is simply an ordinary bunch of keys taken thr6ugh one-quarter of an inch of aluminum, and a watch-chain taken through one quarter of an inch of hard rubber, and in this instance the exposure was three minutes. To show you how the time for exposure has been diminished, here is one that was exposed for forty-five seconds. It is a pocket magnifying-glass and a piece of curved wire taken through. Here is a picture of a normal hand and one of an abnormal hand taken at the City Hospital; the idea was to show the difference between the two. In the abnormal one, one joint of the first finger is missing, and the middle joint is very much distorted. Here is one taken with only a part of the power, but with the plate close to the luminescent screen, with the idea of seeing whether the luminescent substance close to the plate would increase its sensitiveness or not. Apparently it had no effect. Here are photographs of the hands of Mr. and Mrs. Norton, taken with the high-frequency current. There was a diamond ring on Mrs. Norton's finger, but as diamonds are very transparent to the rays, only the gold can be seen in the photograph. After we had succeeded in doing that, we thought we would try a clinched fist and forearm, which is shown on that slide quite clearly; then we tried an elbow joint and the rest of the arm,—this one is pretty dense because of the thickness of the bone, but here is another one of the same elbow from a different point of view, and you can even see the cords without any difficulty at all. This is the hand of a negro who was struck in the wrist by a 22-calibre pistol-ball, and the Röntgen rays show it embedded in the bone, right there, with perfect clearness. Then this next one is the hand of President Walker, of the Institute, which was struck by a splinter of a shell and quite badly broken, so that the bones are very much distorded, and right under the palm of the forefinger is what looks like a little splinter of the shell.

We then changed over to the static influence-machine, and you will notice the improvement in the photographs obtained. They are very much clearer and the time of exposure is very much shorter than with the earlier apparatus.

